# A category contingent aftereffect for faces labelled with different religious affiliation is seen 7 days after adaptation

**DOI:** 10.1177/03010066231100880

**Published:** 2023-05-02

**Authors:** Victoria Foglia, M.D. Rutherford

**Affiliations:** 3710 McMaster University, Canada; 3710 McMaster University, Canada

**Keywords:** visual adaptation, aftereffects, opposing aftereffects, adaptation decay

## Abstract

Visual adaptation occurs after a prolonged exposure to a stimulus. The duration of aftereffects differs across stimuli type, and face aftereffects may be especially long lasting. The current study investigates adaptation decay of category contingent opposing aftereffects. Specifically, we tested whether naïve undergraduate participants’ adaptation to photos of faces with explicit religious labels, differed from that of participants who had adapted to the same faces 7 days previously. We also tested whether 7-day old category-contingent opposing aftereffects interfere with the ability to re-adapt to a new condition. In Session 1, undergraduates made attractiveness preference selections before and after adapting to two groups of distorted faces. Participants then returned 7 days later to re-assess the attractiveness of the same faces. Participants were then adapted to the two groups of faces distorted in the opposite direction. Adaptation strength was stronger in Session 1 than in Session 2, although adaptation strength was not related to pre-adaptation selections. Week-old aftereffects interfered with the creation of aftereffects in the opposite direction 7 days later.

## Face Aftereffects Impact the Perception of Faces After a 1-Week Delay

Visual adaptation consists of a distortion in perception following a prolonged exposure to a stimulus (Clifford & Rhodes, 2005; Webster, 2011). This distortion is called an aftereffect. Adaptation involves a change in the perceptual system and causes reduced activity in the neurons responding to the adapting stimulus (Barlow & Hill, 1963; Bednar & Miikkulainen, 2000). The result is the perception of a neutral stimulus as the visual opposite of the fixated stimulus. For example, after viewing a line that is tilted in one direction, a vertical line will appear as if it is tilted in the opposite direction (Bednar & Miikkulainen, 2000; Gibson, 1933). After viewing a waterfall for several seconds, a nearby stationary rock will appear to move upwards (Barlow & Hill, 1963). Though aftereffects may be misperceptions, they have been frequently used as an experimental technique in understanding perception (see Clifford & Rhodes, 2005; Webster, 2011 for reviews).

The duration of visual aftereffects varies, and in some cases, aftereffects persist for months (Jones & Holding, 1975). Afterimages are a particular type of aftereffect, and afterimage decay has been found to vary. Duration may provide a clue about the neural basis. If the afterimage results from adaptation at the level of a single neuron, the afterimage would be expected to be fleeting. Afterimage durations of days or longer suggest that the effect does not rely on simple cell fatigue but rather an underlying connection between neurons at different levels in the central nervous system (Murch & Hirsch, 1972). Face afterimages may be expected to have a particularly long duration, because the norms-based model of face perception suggests that we identify an individual by comparing a perceived face to a stored face template (Rhodes & Jeffery, 2006). That stored template is, according to the model, influenced by all of the faces one has viewed. It is the averaged, normal face, to which a perceived face is contrasted (Valentine, 1991).

Simple aftereffects have been observed for a variety of visual properties such as color (Gurnsey et al., 1994), motion (Clifford, 2002), contrast (Gibson, 1933), size (Blakemore & Sutton, 1969), texture (Durgin & Proffit, 1996), and shape (Suzuki, 2003). Simple aftereffects have been also observed for more complex visual stimuli, such as faces. For example, face identity aftereffects have been observed when viewing one face leads to another face being perceived as differing from average in the opposite way (Rhodes & Jeffery, 2006). Additionally, aftereffects have been observed for temporary aspects of the face, such as eye gaze direction (Jenkins et al., 2006) and emotion expression (Rutherford et al., 2008; Webster et al., 2004).

Opposing face aftereffects are especially interesting, as the human visual system is thought to create discrete face templates for some social categories, such as sex (Jaquet et al., 2008; Little et al., 2005) or race (Jaquet et al., 2008; Little et al., 2008). Opposing face aftereffects occur when one category of distorted faces is adapted to, and simultaneously another face category which has been distorted in the opposite direction is also adapted to. For example, observers can adapt to contracted male faces and expanded female faces simultaneously. Opposing aftereffects paradigms are often used to test for discrete face templates that encode representations of different social categories of faces. If it is possible to adapt to both face categories in opposite directions, this then suggests that the neural coding for faces in one category is distinct from the neural coding of faces in the other category. Opposing face aftereffects have been observed in adults for race (Jaquet et al., 2008; Little et al., 2008), gender (Jaquet et al., 2008; Little et al., 2005), age, species (Little et al., 2008), and religion (Foglia et al., 2021).

Simple configural face aftereffects (Leopold et al., 2005; Rhodes et al., 2007) and simple identity aftereffects (Rhodes et al., 2007) have shown a decay time course similar to simple visual aftereffects such as tilt (Harris & Calvert, 1989), with decay diminishing within seconds after adaptation. Identity aftereffects have been found to begin to decay as early as 300 ms after adaptation (Leopold et al., 2005). Rapid decay of aftereffects are a challenge when trying to measure the adaptation during a test session that lasts several minute, so some face adaptation paradigms include top-up adapting faces during post-adaptation test sessions to maintain the adaptation (Anzures et al., 2009; Rhodes et al., 2003). Gaze aftereffects have been found to be measurable several minutes after adaptation (Kloth & Schweinberger, 2008), as well as up to 24 h after adaptation (Kloth & Rhodes, 2016). Expression aftereffects have been estimated to fully decay within 9 h after adaptation (Burton et al., 2016).

The adaptation persistence of simple face aftereffects increases as a function of the length of adaptation duration and decreases within the test duration (Leopold et al., 2005; Rhodes et al., 2007). Simple face aftereffects following adaptation to geometrically distorted famous faces have been shown to be measurable after a 1-week delay, after participants saw each image 15 times, for either 2, 3, or 4 s duration (Carbon & Ditye, 2012). Opposing aftereffects were not tested in these studies.

Color aftereffects have been observed to persist weeks after adaptation (Neitz et al., 2002). The McCollough effect, a complex, orientation-dependent color aftereffect, has been seen to persist for nearly 3 months, although this persistence is degraded by retesting (Jones & Holding, 1975). These results suggest that time course of decay may vary across the stimuli being adapted to.

The current study was designed to test whether any persistent opposing aftereffects influence a subsequent adaptation session, and to test participants’ ability to adapt to facial distortions in a different direction 7-day after the initial face adaptation session. Opposing face aftereffects may be especially long if such aftereffects involve updating discrete face templates. This study employs the same category-contingent opposing aftereffects paradigm as used in Experiment 2 of Foglia et al. (2021). In the current study, participants completed two adaptation sessions. In the first, they adapted to differently distorted “Christian” and “Muslim faces” as defined in the Methods section (e.g., Christian contracted/Muslim expanded OR Christian expanded/Muslim contracted). Then, 7 days later participants adapted to each group distorted in the opposite direction from what they saw in Session 1, and adaptation was measured again. If decay of the aftereffects occurs rapidly, then after 7 days participants should perform similarly to the naïve participants in Session 1. However, if the adaptation persists for 7 days, effects from Session 1 may interfere with the ability to adapt to Session 2.

## Methods

### Photo Stimuli

**Figure 1.**
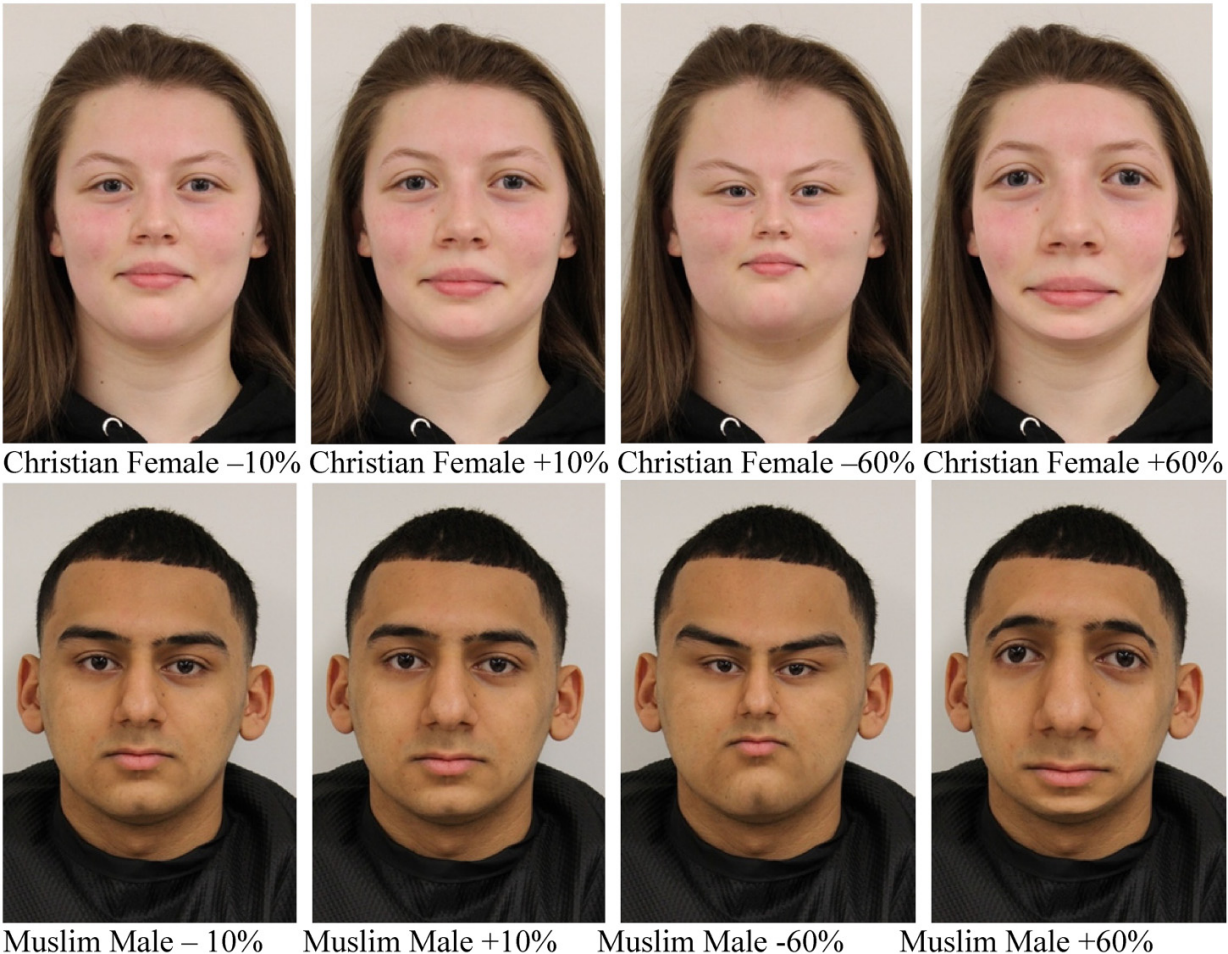
Stimuli samples of the faces used in Experiment 2. Pre- and post-adaptation phases consisted of presenting +/−10% faces. Adaptation consisted of +/−60% faces.

Undergraduate students were recruited through emails and social networking groups for McMaster University as face models. The recruitment emails asked for Christian and Muslim models and consisted of a set demographic questions about their and their family's religious heritage. All models self-identified as Christian or Muslim (see Foglia et al., 2021, Table 1 for additional demographic information). Models self-identified religious identity determined category membership as “Christian” or “Muslim” for this study. Photo stimuli were validated (see Foglia et al., 2021, Experiment 1). This confirmed that all stimuli in one group were perceived as visually distinct from all stimuli in the other, see sample stimuli in Supplemental Material. The terms “Muslim” and “Christian” will be used throughout this paper to describe these two groups (Figure 1).

**Table 1.** Pre- and post-adaptation contracted face selections from Session 1 & Session 2.

Group & session	Adaptation condition	Pre-adaptation Christian selections	Pre-adaptation Muslim selections	Post-adaptation Christian selections	Post-adaptation Muslim selections
Group 1 Session 1: Adapted to Christian expanded/Muslim contracted	*M*	15.28	16.39	17.06	19.22
	*SD*	4.32	3.84	3.54	3.34
Group 2 Session 1: Group adapted to Christian contracted/Muslim expanded	*M*	15.19	15.19	18.63	17.19
	*SD*	3.94	4.79	3.81	5.22
Group 1 Session 2: Adaptation to Christian contracted/Muslim expanded	*M*	18.06	18.78	18.72	18.67
	*SD*	3.44	4.61	3.54	4.52
Group 2 Session 2: Adaptation to Christian expanded/Muslim contracted	*M*	14.31	17.56	16.31	17.38
	*SD*	5.94	5.89	6.65	6.91

**Figure 2.**
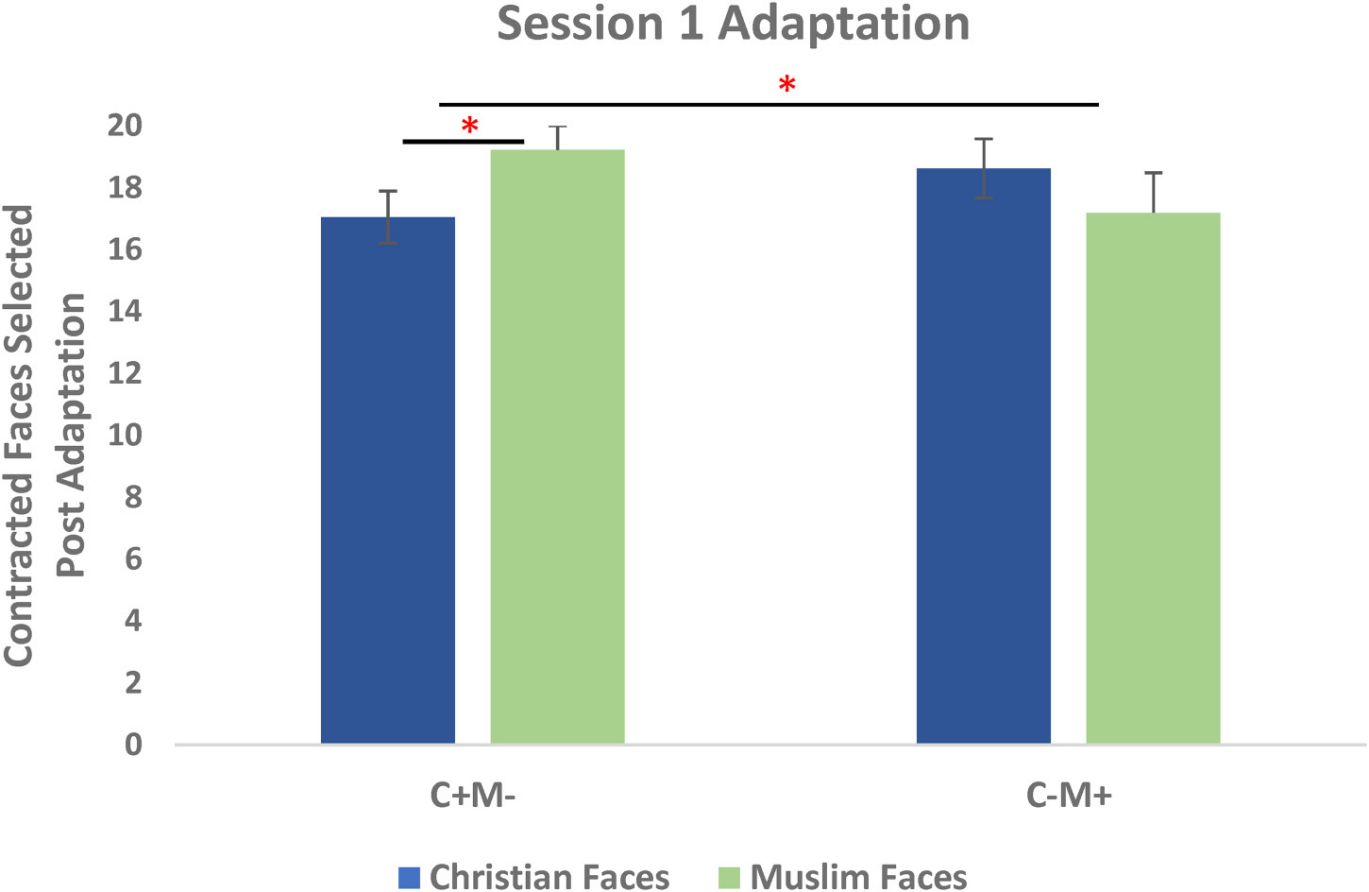
Mean contracted faces selected post-adaptation during Session 1. Significant opposing aftereffects were observed for participants who were first trained on expanded Christian and contracted Muslim images (C+M−) and approached significance for the remainder of the participants (C−M+).

Eighteen photos were used in the current study, and all participants viewed the same models across the two sessions. Participants viewed 12 faces, 6 Christian and 6 Muslim during pre- and post-adaptation testing. Half of the models in each group were male, and half were female. These same faces were used for pre- and post-adaptation testing regardless of adaptation condition or session. Participants then adapted to an additional 6 faces, 3 Christian and 3 Muslim. The same models were used regardless of adaptation condition or session, but the stimuli differed based on the direction of distortion (expanded or contracted). These faces had supported opposing aftereffects in a previous study, but only if explicit religious labels via audio descriptions accompanied the presentation of the photograph (see Foglia et al., 2021; Figure 2 for difference in adaptation with and without religious explicit audio descriptions).

Each original photo was expanded by +10 and +60 and compressed by −10 and −60 using the spherize function on Adobe Photoshop CS, yielding four manipulated images per model. For more information on stimulus development, see Foglia et al. (2021). For samples of the stimulus sets, see Supplemental Material.

### Audio Stimuli

In the pre-adaptation phases, images were presented with audio recordings. Audio recordings were those used in Experiment 2 of Foglia et al. (2021), and provided categorical information about the models. See Foglia et al. (2021) for more information on the development of the audio and visual stimuli.

### Participants

Thirty-six McMaster University students participated in this study. Two participants failed to complete both sessions and were therefore excluded from analyses. Analyses included 34 participants (16 male; 18 female) ages ranged from 18 to 23 (*M* = 18.6, *SD* = 1.21). The sample size was selected to replicate the number of participants per adaptation group as in Experiment 2 by Foglia et al. (2021) but ultimately had a smaller sample size. Participants received course credit for their time. Informed consent was obtained from all participants. Ethics permission was obtained from the McMaster University Research Ethics Board. All work was carried out in accordance with The Code of Ethics of the World Medical Association (Declaration of Helsinki) for experiments involving humans.

### Procedure

Participants were adapted in one of two adaptation session orders: They were either adapted to Christian expanded and Muslim contracted faces in the first session and contracted Christian and expanded Muslim in the second session or vice versa. All participants completed both adaptation conditions, with the 2 sessions scheduled 7 days apart. In each session, participants completed an identical procedure using a 15-inch ASUS laptop with the screen approximately 40 cm from the participant's eyes.

### Session 1 Procedure

At the beginning of Session 1, participants were given a description of the task and signed a consent form.

#### Pre-adaptation

Participants viewed 12 pairs of faces—6 from each group, 4 times each in a randomized order. Each pair of images depicted the same model with one face expanded by 10% and the other contracted by 10% and participants were asked which of the two faces they found more attractive in order to measure their baseline preference for contracted faces before adaptation. For each model, half of the trials showed the expanded face was on the left, and the other half the right. An audio clip played along with each face pair, stating the name of the person depicted. Each pair was presented for 2 s, followed by a prompt screen instructing the participants to select via keypress which face they found more attractive.

#### Adaptation

In the adaptation phase participants fixated 60% distorted faces that were either contracted Christian and expanded Muslim faces or vice versa, depending on condition. Participants viewed three images from each religious group, one at a time, three times each. Faces were presented in a randomized order for 7 s per face with a 500 ms inter-stimulus interval. Each face was paired with a character description audio clip.

#### Post-adaptation

The post-adaptation procedure was nearly identical to the pre-adaptation procedure. All face pairs were the same, and to maintain any adaptation, six top-up faces from the adaptation phase were presented for 1 s each in a randomized order, between the attractiveness ratings of the face pairs.

### Session 2 Procedure

All participants returned to the lab for a second session 7 days after the first session. The experimental task was explained again, and participants completed a second consent form.

#### Pre-adaptation

Participants underwent the same pre-adaptation session procedure as they had completed 7 days prior.

#### Adaptation

The second sessions adaptation phase was identical to the first session adaptation phase, except that the direction of each participant's distortion was reversed, and the stimulus order was re-randomized.

#### Post-adaptation

After the second adaptation phase, participants underwent the same post-adaptation session procedure as they had completed 7 days prior.

#### Demographic Questionnaires

Upon completion of the experimental tasks of Session 2, participants completed a questionnaire asking for their age, gender, country of birth, family's religious background, and the religion they practiced, if any. Participants were also asked to indicate how often they practiced their religion on a scale from 1 (never) to 5 (very frequently). Finally, participants were debriefed.

## Results

There was no significant differences in post-adaptation selections in Session 1 based on model gender, (*t(*32) = 0.60, *p* = 0.55). Since selections did not differ by gender of model, and gender was not the social category of interest in the present study, model gender was not included within the model. Adaptation to Christian and Muslim faces was examined using the same opposing-aftereffects analyses as Foglia et al. (2021).

In order to test whether face aftereffects were created in Session 1, a 2 (Adaptation Condition) by 2 (Face Group) repeated measures ANOVA was conducted using the number of trials in which a contracted face was preferred, measured post-adaptation during Session 1. A Shapiro-Wilk's test showed that the post-adaptation scores were normally distributed for both Christian (*W* = 0.200, *p* *=* .67) & Muslim faces (*W* = 0.200, *p* *=* .38). There was a significant interaction between adaptation condition and Face Group (*F*(1, 32) = 9.88, *p* = .004). There was no main effect of Adaptation Condition (*F*(1, 32) = 0.035, *p* = .854) or Face Group (*F*(1, 32) = 0.404, *p* = .529) (see Figure 2) (See Table 1 for group means and standard deviations.).

To test for opposing aftereffects, we conducted one-tailed paired *t*-tests for each adaptation condition separately. For participants trained on expanded Christian faces and contracted Muslim faces, significant opposing aftereffects were observed (*t*(17) = −3.11, *p* = .003), with more preference for contracted Muslim faces (*M* = 19.22, *SD* = 3.54), than contracted Christian faces (*M* = 17.06, *SD* = 4.34) as expected. For the other Adaptation Condition, opposing aftereffects approached significance (*t*(16) = 1.54, *p* = .07), with more preference for contracted Christian faces (*M* = 18.63, *SD* = 3.81), than contracted Muslim faces (*M* = 17.19, *SD* = 5.21) as expected. Opposing aftereffects were created in Session 1 among naïve participants (see Figure 2).

Participants returned after 7 days and were adapted to the opposite adaptation condition. Aftereffects were assessed to determine if participants were capable of re-adapting to a second condition in the opposite direction after a 7-day decay from the prior condition's direction.

**Figure 3.**
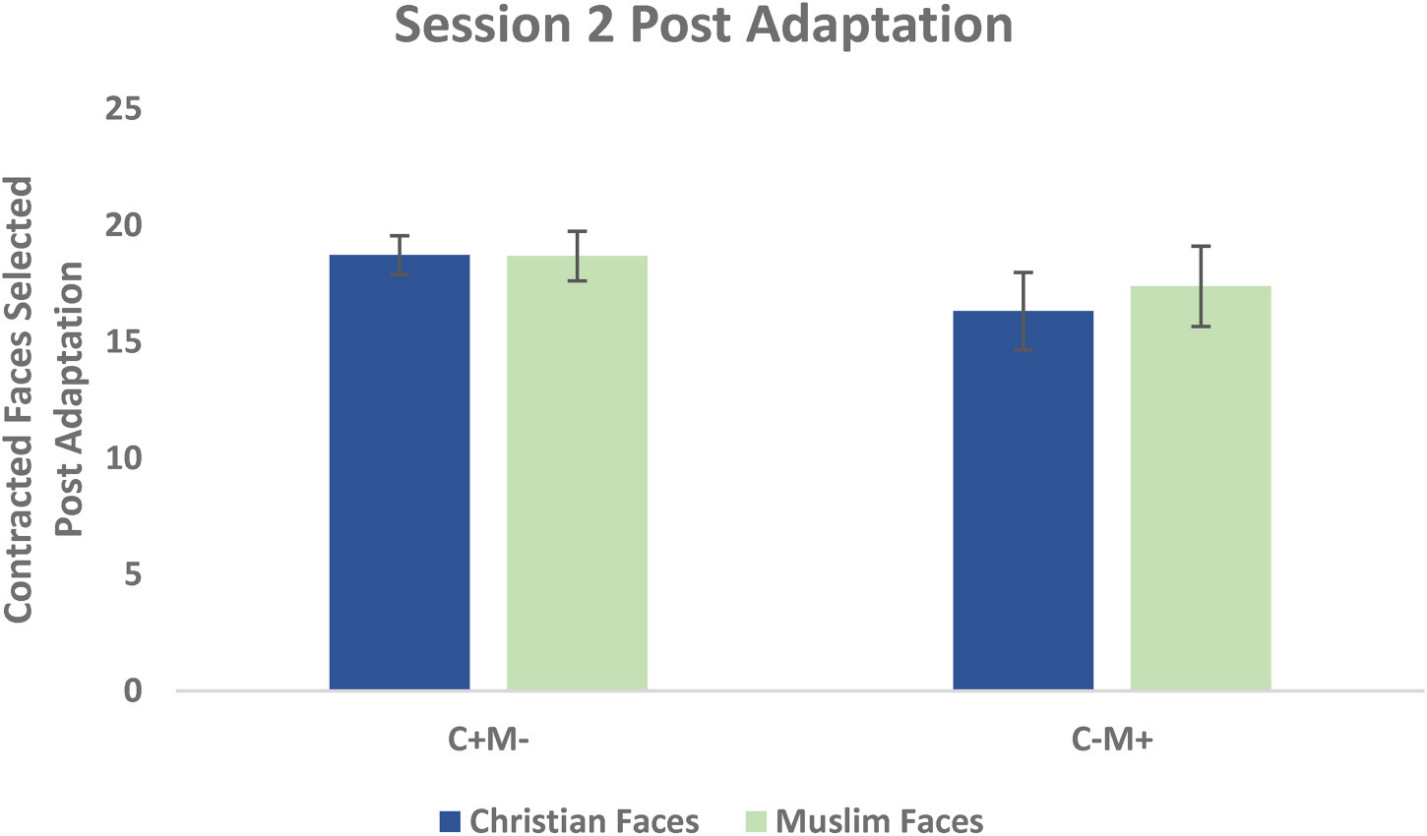
Mean contracted faces selected post-adaptation Session 2 for the two adaptation conditions. Opposing aftereffects were not observed.

A 2 (Adaptation Condition) by 2 (Face Group) repeated measures ANOVA was conducted using the number of trials in which a contracted face was preferred, measured post-adaptation after adaptation in Session 2. A Shapiro-Wilk's test showed that the post-adaptation scores were normally distributed for both Christian (*W* = 0.196, *p* *=* .86) and Muslim faces (*W* = 0.200, *p* *=* .86). No significant main effects were found for Face Group (*F*(1, 32) = 0.64, *p* = .43), for Adaptation Condition (*F*(1, 32) = 1.08, *p* = .31), nor was there an interaction (*F*(1, 32) = 0.79, *p* = .38) suggesting that 7 days after the original adaptation, opposing aftereffects in the opposite adaptation direction could not be created (see Figure 3).

In order to test whether face aftereffects persisted until pre-adaptation Session 2, a 2 (Adaptation Condition) by 2 (Face Group) repeated measures ANOVA was conducted using the number of trials in which a contracted face was preferred before adaptation in Session 2. There was no significant interaction between Adaptation Condition and Face Group (*F*(1, 32) = 0.322, *p* = .575) or main effect of Face Group (*F*(1, 32) = 0.322, *p* = .575). There was a main effect of Adaptation Condition (*F*(1, 32) = 6.150, *p* = .019). Those trained on expanded Christian and contracted Muslim in Session 1 preferred more contracted faces overall faces (*M* = 17.19, *SD* = 3.904) when trained on contracted Christian and expanded Muslim in Session 2 (*M* = 15.188, *SD* = 4.969). As there was no significant interaction between Adaptation Condition and Face Group, pre-adaptation score did not differ based on face groups across adaptation conditions, and opposing aftereffects were not observed at the beginning of Session 2.

To examine the strength of naïve participants adaptation compared to their adaptation a week later as experienced participants, change scores were compared across sessions 1 and 2. Participant's change in preference for contracted faces scores were the difference in the number of contracted faces chosen before and after adaptation in each session, collapsed across face group to get an overall adaptation strength. Half of the adaptation scores were reverse coded since adaptation was expected to be in opposite directions across the adaptation conditions.

**Figure 4.**
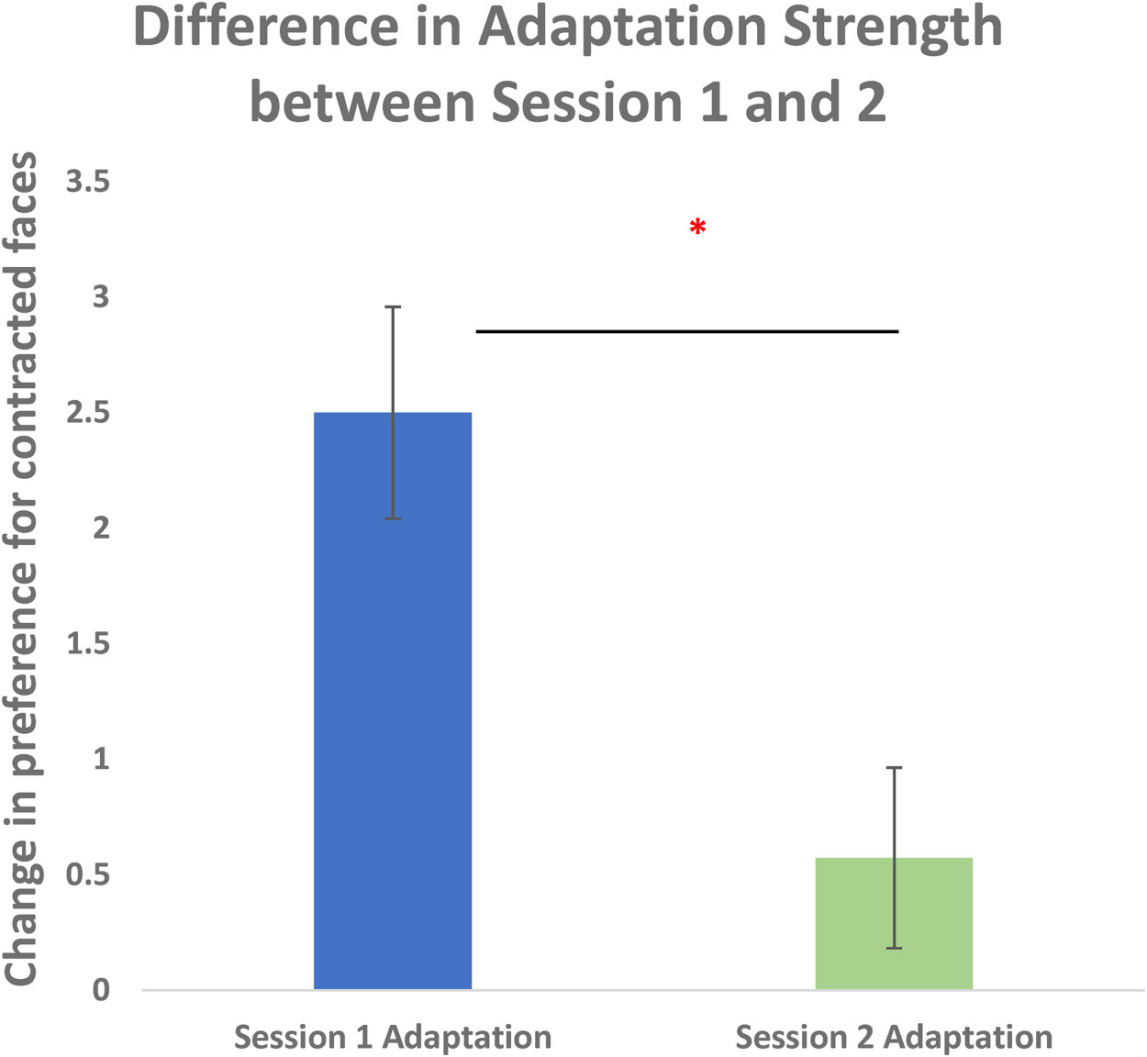
Difference in change in preference for contracted faces from Session 1 to Session 2. Participants adapt significantly more in Session 1 than in Session 2.

A 2 (first Adaptation Condition) by 2 (session: Session 1 or Session 2) repeated measures ANOVA was conducted using participants’ change scores as the dependent variable. A Shapiro-Wilk's test showed that the change scores were normally distributed for both Christian (*W* = 0.984, *p* *=* .979) and Muslim faces (*W* = 0.972, *p* *=* .842). A main effect of adaptation session time was observed (*F*(1, 62) = 12.12, *p* = .001). Participants adapted more strongly when they were naïve, in Session 1 (*M* = 2.45, *SD* = 3.78) than they did when they were experienced, in Session 2 (*M* = 0.55, *SD* = 3.23) (see Figure 4). There was no significant main effects of participant group (*F*(1, 62) = 0.76, *p* = .39) nor any interaction between participant group and adaptation session (*F*(1, 62) = 0.02, *p* = .90).

Adaptation decay was also examined for both adaptation groups separately. For those who adapted to expanded Christian and contracted Muslim faces in Session 1, there was a significant difference in the number of contracted Muslim faces selected pre-adaptation Session 2 compared to pre-adaptation Session 1, consistent with the distortion direction of adaptation 7 days prior. Participants selected more contacted Muslim faces pre-adaptation Session 2 (*M* = 18.78, *SD* = 21.24) than they did pre-adaptation in Session 1, 7 days prior (*M* = 16.39, *SD* = 14.72) (*t*(17) = −2.373, *p* = .015). Participants also preferred significantly more contracted Christian faces pre-adaptation Session 2 (*M* = 18.056, *SD* = 11.820) than they did in pre-adaptation Session 1 (*M* = 15.278, *SD* = 18.683) which is opposite to what would be predicted given their adaptation condition (*t*(17) = −2.909, *p* = .005).

For those who trained to contracted Christian and expanded Muslim in Session 1, there was no significant difference between participants pre-adaptation selections of contracted Christian faces between Session 1 and Session 2 (*t*(16) = 0.599, *p* = .279). Participants also preferred more contracted Muslim faces in pre-adaptation Session 2 (*M* = 17.563, *SD* = 34.663) than in pre-adaptation Session 1 (*M* = 15.186, *SD* = 22.963), which is opposite to what would be predicted for this adaptation condition (*t*(16) = −2.169, *p* = .023).

Some research showing adaptation to distorted faces have reported different adaptation strengths to contracted versus expanded faces (Foglia et al., 2021). Therefore, we tested the strength of adaptations induced by fixating contracted faces and adaptations induced by fixating expanded faces separately across Session 1 and Session 2. The adaptation strength consisted of change scores from participants who adapted to Christian contracted faces in the first session combined with participants who adapted to Muslim contracted faces in the first session, while the converse was true for measures of adaptation to expanded faces.

**Figure 5.**
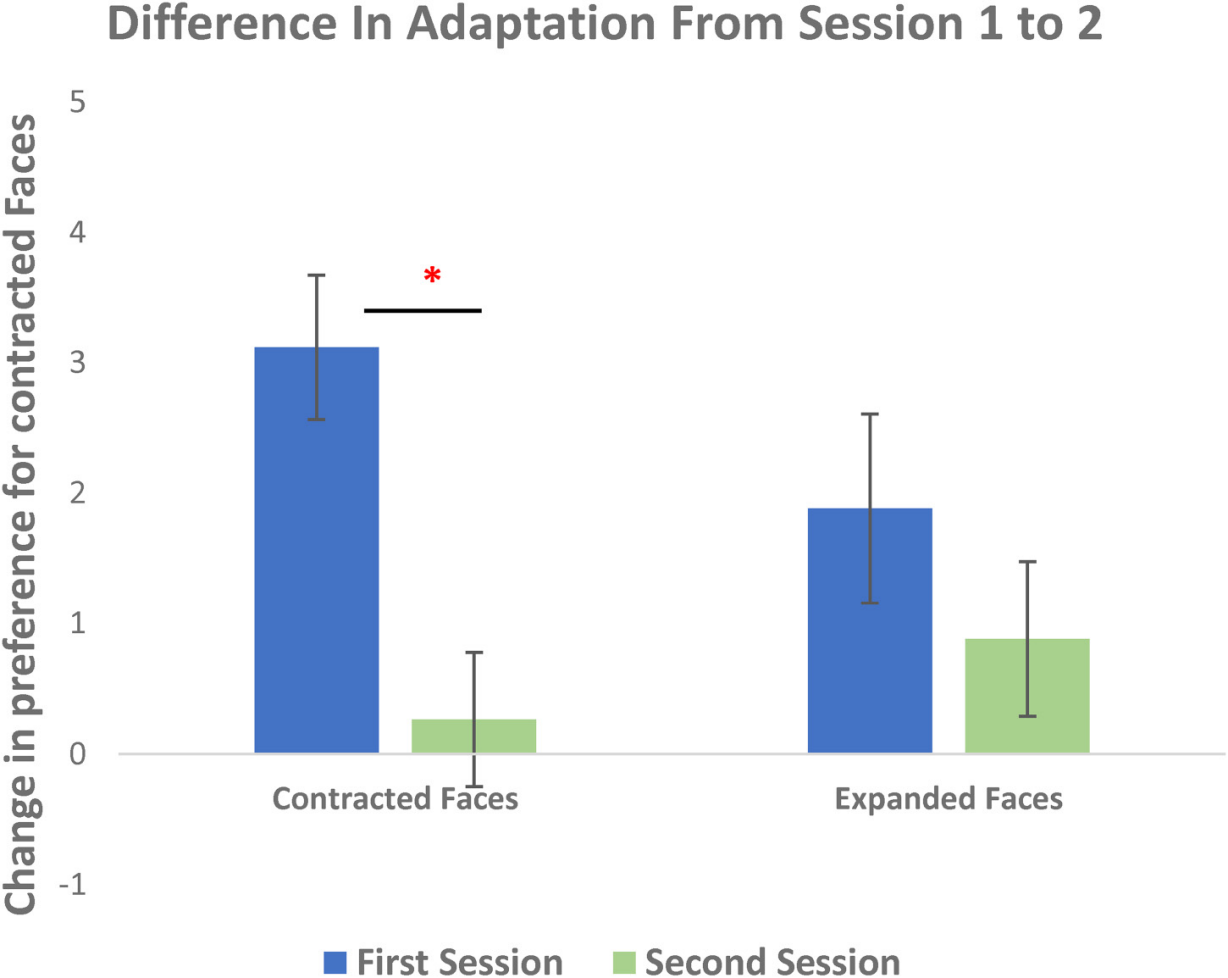
Change in preference for contracted faces for all contracted faces collapsed and expanded faces collapsed from Session 1 to Session 2. There is a significant difference in adapting in the expected distortion direction to contracted faces from Session 1 to Session 2, but not expanded faces*.*

A repeated measures ANOVA was conducted to compare responses to contracted and expanded faces across sessions. There was more change in preference for contracted faces in Session 1 (*M* = 3.12, *SD* = 3.23) than Session 2 (*M* = 0.26, *SD* = 3.01) and this difference was significant (*F*(1, 67) = 14.22, *p* = <.001). However, there was no significant difference in the change in preference for expanded faces between Session 1 (*M* = 1.88, *SD* = 4.25) and Session 2 (*M* = 0.88, *SD* = 3.45) (*F*(1, 67) = 1.14, *p* = .289) (see Figure 5). Therefore, adaptation to contracted faces but not expanded faces was interfered with 7 days after adapting to a different distortion.

To test whether an individual's adaptation strength from Session 1 influenced perception at the beginning of Session 2, correlations were computed between the adaptation strength calculated in Session 1 (change in the number of contracted faces selected from pre-to-post-adaptation Session 1) and pre-adaptation Session 2 selections. Positive correlations between adaptation strength in Session 1 and pre-adaptation selections in Session 2 were predicted as contracted faces preference would remain consistent if there is little adaptation decay. However, no significant correlations were observed in the expected direction.

For participants who adapted to contracted Christian and expanded Muslim faces during their first session, a negative correlation approached significance between the change in adaptation strength for Christian faces in Session 1 compared to pre-adaptation Session 2 (*r*(16) = −.473, *p* = .064), two-tailed, though this is in the opposite direction of that would be expected. No correlation was observed between the change in adaptation strength for Muslim faces in Session 1 compared to pre-adaptation Session 2 (*r*(16) = −.400, *p* = .125).

For participants who adapted to expanded Christian and contracted Muslim faces, no significant correlations were observed between strength of adaptation in Session 1 and pre-adaptation Session 2 for either Christian (*r*(18) = −.0336, *p* = .173) or Muslim (*r*(18) = .34, *p* = .822) faces.

## Discussion

The purpose of the current study was to test whether naïve participants’ adaptation to faces differs from that of participants who had adapted to faces 7 days previously, and whether experience with face adaptation interfered with an observer's ability to re-adapt to faces that were distorted in the opposite direction. Participants adapted to faces that were either contracted or expanded in Session 1, and then returned to the lab a week later, re-evaluating the same faces. Although significant opposing aftereffects were observed in Session 1, reversed opposing aftereffects were not evident 7 days later in Session 2. Additionally, participants adapted more strongly in Session 1 than in Session 2, presumably because their previous adaptation interfered with subsequent adaptation. This interference suggests a week-long persistence of adaptation to contracted faces. Similarly, color aftereffects have also been found to persist weeks after adaptation (Delahunt et al., 2004; Neitz et al., 2002) including the McCollough effect, a complex orientation-contingent color aftereffect that has been shown to persist nearly 3 months in some circumstances (Jones & Holding, 1975).

A reversal of the adaptation was not evident in Session 2. When participants returned for Session 2, they saw faces that had been manipulated in the opposite direction as the faces seen 7 days prior. Opposing aftereffects did not occur after this second adaptation, indicating that the adaptation from Session 1 not only persisted 7 days later, but interfered with the ability to adapt in the opposite direction. Previously opposing aftereffects for this set of stimuli have been observed in naive participants both here and in previous research (Foglia et al., 2021). Therefore, adapting to a previous condition interfered with the ability to re-adapt to the same faces 7 days later.

Adaptation to contracted faces was found to decay differently, and more slowly than to expanded faces. This duration difference has not been previously reported. Decay has been reported to begin immediately after adaptation to facial expressions (Burton et al., 2016), face identity (Leopold et al., 2005) and simple figural face aftereffects (Rhodes et al., 2007), with the length of decay depending on length of fixation during adaptation. For example, Burton et al. (2016) has estimated that expression aftereffects would be extinct within hours of adaptation following a maximum adaptation of 16 s. Leopold et al. (2005) tested aftereffects after an adaptation of 1, 2, 4, 8 or 16 s, and they report logarithmic accumulation with exposure to the adapting stimulus, exponential decay over the test period. Rhodes et al. (2007) included adaptation durations of up to 16 s and again reported a relationship between exposure and aftereffect persistence. Carbon and Ditye reported a week-long persistence of a simple aftereffect following 15 exposures (ranging from 2 to 4 s) to a distorted famous face (Carbon & Ditye, 2012). In the current study, adaptation involved a total of 126 s of exposure to distorted face, including 63 s for each distortion direction. Perhaps the repeated and cumulative visual adaptation in the current experiment contributed to the persistence of the aftereffect.

The relatively long duration of opposing face aftereffects is consistent with the idea that these effects cannot be due to simple cell fatigue. These aftereffects more likely involve connection between neurons at different levels in central nervous system (Murch & Hirsch, 1972). Furthermore, persistent effects after the inducement of opposing aftereffects would be predicted by a norm-based coding model, which posits that face perception relies on stored templates that represent the average of all faces one has seen. These templates can be updated with new faces are seen, and discrete templates for different face categories can be updated independently (e.g., see Rhodes & Jeffery, 2006).

Several other aftereffects have been found to have a short time course (Harris & Calvert, 1989; Krauskopf, 1954; Magnussen & Johnsen, 1986; Wolfe, 1984). For example, contrast aftereffects that form after brief visual exposure begin to decay within seconds after adaptation, but adaptations resulting from longer fixation decay more slowly (Harris & Calvert, 1989). Tilt aftereffects decay within 30 min after adaptation (Magnussen & Johnsen, 1986).

Adaptation strength was found to be stronger in Session 1, when participants were naïve, compared to Session 2, when participants had previously adapted to distorted faces. When investigated further, decay was particularly slow for contracted faces. However, the correlational analyses between adaptation strength in Session 1 and pre-adaptation selections in Session 2 were not significant or not consistent in the expected direction. Adaptation strength in Session 1 would be expected to be related to pre-adaptation selections in Session 2 if there was a long-lasting decay of adaptation. Further studies are needed to create a fuller description of the decay trajectory.

We know from previous work that the explicit religious label played a role in the creation of the opposing aftereffects seen here. Given the same face images and the same adaptation treatment but character descriptions that lack any mention of religious group membership, opposing aftereffects were not seen (Foglia et al., 2021). Others have reported opposing aftereffects in adults across race and gender categories without the use of audio labels (Jaquet et al., 2008; Little, et al., 2005; Little et al., 2008). Foglia et al. (2021) reported that opposing aftereffects were induced only among participants who heard religious labels and explicit religious descriptions. Physical characteristics of the two sets of images based on religion, ethnicity, or country of birth were not distinct enough to evoke an aftereffect on their own. In contrast, studies showing opposing aftereffects across race and gender categories have induced these aftereffects without making explicit the race or gender of the models (Jaquet et al., 2008; Little et al., 2005; Little et al., 2008). Perhaps the categories of race and religion are not psychologically equivalent. Religious categories may lack a visually perceivable social distinction until the social information is explicitly labelled.

### Limitations and Future Directions

The current study revealed that there was interference in re-adapting to contracted faces 7 days after the first adaptation but leaves open the question of how long opposing aftereffects ultimately last. Future studies could re-test participants at multiple timepoints to describe the trajectory of adaptation decay over time. More specifically, future studies could examine when we observe complete decay, and how this effects the relationship between Session 1 and Session 2.

Additionally, whether the effects of the current study were modulated by the social category observed could be further examined. Opposing aftereffects have previously been observed for several other social categories, such as sex and race (Jaquet et al., 2008; Little et al., 2005; Little et al., 2008). As time decay for aftereffects vary based on the stimuli presented (Burton et al., 2016; Harris & Calvert, 1989; Kloth & Rhodes, 2016; Kloth & Schweinberger, 2008; Magnussen & Johnsen, 1986; Neitz et al., 2002), opposing aftereffects decays across other social categories should not be assumed to be the same. Future studies could test for persistence of opposing aftereffects across categories defined by age and sex to examine the length of persistence and if there are differences based on the direction of the face.

The current study specifically focused on adaptation decay within an opposing aftereffects paradigm, but it leaves open questions about whether difference in decay were due to adaptation alone, or this specific form of adaptation where social categorization with two opposing face categories being provoked. Future studies could compare simple versus opposing adaptation decay (e.g., adaptation to 1 category of faces or 2 opposing categories) to determine how long simple adaptation lasts, and how this might differ from opposing aftereffects.

### Conclusion

The purpose of the current study was to explore whether naïve participants’ adaptation to faces differs from that of participants who had adapted to faces 7 days previously, and whether the persistence of previous aftereffects interferes with the creation of aftereffects in the opposite direction. The first adaptation session impacted participants’ ability to adapt during the second session. Seven days after the first adaptation, participants were unable to re-adapt to faces distorted in the opposite direction. The persistence of week-old aftereffects interfered with the creation of aftereffects in the opposite direction 7 days later.

## Supplemental Material

sj-docx-1-pec-10.1177_03010066231100880 - Supplemental material for A category contingent aftereffect for faces labelled with different religious affiliation is seen 7 days after adaptation Supplemental material, sj-docx-1-pec-10.1177_03010066231100880 for A category contingent aftereffect for faces labelled with different religious affiliation is seen 7 days after adaptation by Victoria Foglia and M.D. Rutherford in Perception

## References

[bibr1-03010066231100880] Anzures G. Mondloch C. J. Lackner C. (2009). Face adaptation and attractiveness aftereffects in 8-year-olds and adults. Child Development, 80, 178–191. 10.1111/j.1467-8624.2008.01253.x 19236400

[bibr2-03010066231100880] Barlow H. B. Hill R. M. (1963). Evidence for a physiological explanation of the waterfall phenomenon and figural after-effects. Nature, 200, 1345–1347. 10.1038/2001345a0 14098503

[bibr3-03010066231100880] Bednar J. A. Miikkulainen R. (2000). Tilt aftereffects in a self-organizing model of the primary visual cortex. Neural Computation, 12, 1721–1740. 10.1162/089976600300015321 10935924

[bibr4-03010066231100880] Blakemore C. Sutton P. (1969). Size adaptation: A new aftereffect. Science, 166, 245–247. 10.1126/science.166.3902.245 5809598

[bibr5-03010066231100880] Burton N. Jeffery L. Bonner J. Rhodes G. (2016). The timecourse of expression aftereffects. Journal of Vision, 16, 1–1. 10.1167/16.15.1 27918785

[bibr6-03010066231100880] Carbon C. C. Ditye T. (2012). Face adaptation effects show strong and long-lasting transfer from lab to more ecological contexts. Frontiers in Psychology, 3, 3. 10.3389/fpsyg.2012.00003 22291676 PMC3264890

[bibr7-03010066231100880] Clifford C. W. (2002). Perceptual adaptation: Motion parallels orientation. Trends in Cognitive Sciences, 6, 136–143. 10.1016/S1364-6613(00)01856-8 11861192

[bibr8-03010066231100880] Clifford C. W. G. Rhodes G. (2005). Fitting the mind to the world: Adaptation and after-effects in high-level vision. OUP Oxford.

[bibr9-03010066231100880] Delahunt P. B. Webster M. A. Ma L. Werner J. S. (2004). Long-term renormalization of chromatic mechanisms following cataract surgery. Visual Neuroscience, 21, 301–307. 10.1017/S0952523804213025 15518204 PMC2633455

[bibr10-03010066231100880] Durgin F. H. Proffit D. R. (1996). Visual learning in the perception of texture: Simple and contingent aftereffects of texture density. Spatial Vision, 9, 423–474. 10.1163/156856896X00204 8774089

[bibr11-03010066231100880] Foglia V. Mueller A. Rutherford M. D. (2021). An explicit religious label impacts visual adaptation to Christian and Muslim faces. Religion, Brain & Behavior, 11(3), 261–280.

[bibr12-03010066231100880] Gibson J. J. (1933). Adaptation, after-effect and contrast in the perception of curved lines. Journal of Experimental Psychology, 16, 1–31. 10.1037/h0074626

[bibr13-03010066231100880] Gurnsey R. Bryden P. J. Humphrey G. K. (1994). An examination of colour-contingent pattern aftereffects. Spatial Vision, 8, 77–94. 10.1163/156856894X00251 8049171

[bibr14-03010066231100880] Harris J. P. Calvert J. E. (1989). Contrast, spatial frequency and test duration effects on the tilt aftereffect: Implications for underlying mechanisms. Vision Research, 29, 129–135. 10.1016/0042-6989(89)90179-X 2773330

[bibr15-03010066231100880] Jaquet E. Rhodes G. Hayward W. G. (2008). Race-contingent aftereffects suggest distinct perceptual norms for different race faces. Visual Cognition, 16, 734–753. 10.1080/13506280701350647

[bibr16-03010066231100880] Jenkins R. Beaver J. D. Calder A. J. (2006). I thought you were looking at me: Direction-specific aftereffects in gaze perception. Psychological Science, 17, 506–513. 10.1111/j.1467-9280.2006.01736.x 16771801

[bibr17-03010066231100880] Jones P. D. Holding D. H. (1975). Extremely long-term persistence of the McCollough effect. Journal of Experimental Psychology: Human Perception and Performance, 1, 323–327. 10.1037/0096-1523.1.4.323 1185119

[bibr18-03010066231100880] Kloth N. Rhodes G. (2016). Gaze direction aftereffects are surprisingly long-lasting. Journal of Experimental Psychology: Human Perception and Performance, 42, 1311–1319. 10.1037/xhp0000182 26962845

[bibr19-03010066231100880] Kloth N. Schweinberger S. R. (2008). The temporal decay of eye gaze adaptation effects. Journal of Vision, 8, 4–4. 10.1167/8.11.4 18831598

[bibr20-03010066231100880] Krauskopf J. (1954). The magnitude of figural after-effects as a function of the duration of the test-period. The American Journal of Psychology, 67, 684–690. 10.2307/1418491 13228729

[bibr21-03010066231100880] Leopold D. A. Rhodes G. Müller K.-M. Jeffery L. (2005). The dynamics of visual adaptation to faces. Proceedings of the Royal Society B: Biological Sciences, 272, 897–904. 10.1098/rspb.2004.3022 PMC1564098 16024343

[bibr22-03010066231100880] Little A. C. DeBruine L. M. Jones B. C. (2005). Sex-contingent face after-effects suggest distinct neural populations code male and female faces. Proceedings of the Royal Society B: Biological Sciences, 272, 2283–2287. 10.1098/rspb.2005.3220 PMC1560190 16191641

[bibr23-03010066231100880] Little A. C. DeBruine L. M. Jones B. C. Waitt C. (2008). Category contingent aftereffects for faces of different races, ages and species. Cognition, 106, 1537–1547. 10.1016/j.cognition.2007.06.008 17707364

[bibr24-03010066231100880] Magnussen S. Johnsen T. (1986). Temporal aspects of spatial adaptation. A study of the tilt aftereffect. Vision Research, 26, 661–672. 10.1016/0042-6989(86)90014-3 3739240

[bibr25-03010066231100880] Murch G. M. Hirsch J. (1972). The McCollough effect created by complementary afterimages. The American Journal of Psychology, 85, 241–247. 10.2307/1420664 5040619

[bibr26-03010066231100880] Neitz J. Carroll J. Yamauchi Y. Neitz M. Williams D. R. (2002). Color perception is mediated by a plastic neural mechanism that is adjustable in adults. Neuron, 35, 783–792. 10.1016/S0896-6273(02)00818-8 12194876

[bibr27-03010066231100880] Rhodes G. Jeffery L. (2006). Adaptive norm-based coding of facial identity. Vision Research, 46, 2977–2987. 10.1016/j.visres.2006.03.002 16647736

[bibr28-03010066231100880] Rhodes G. Jeffery L. Clifford C. W. Leopold D. A. (2007). The timecourse of higher-level face aftereffects. Vision Research, 47, 2291–2296. 10.1016/j.visres.2007.05.012 17619045

[bibr29-03010066231100880] Rhodes G. Jeffery L. Watson T. L. Clifford C. W. G. Nakayama K. (2003). Fitting the mind to the world: Face adaptation and attractiveness aftereffects. Psychological Science, 14, 558–566. 10.1046/j.0956-7976.2003.psci_1465.x 14629686

[bibr31-03010066231100880] Rutherford M. D. Chattha H. M. Krysko K. M. (2008). The use of aftereffects in the study of relationships among emotion categories. Journal of Experimental Psychology: Human Perception and Performance, 34, 27–40. 10.1037/0096-1523.34.1.27 18248138

[bibr32-03010066231100880] Suzuki S. (2003). Attentional selection of overlapped shapes: A study using brief shape aftereffects. Vision Research, 43, 549–561. 10.1016/S0042-6989(02)00683-1 12595000

[bibr33-03010066231100880] Valentine T. (1991). A unified account of the effects of distinctiveness, inversion, and race in face recognition. The Quarterly Journal of Experimental Psychology, 43(2), 161–204. 1866456 10.1080/14640749108400966

[bibr34-03010066231100880] Webster M. A. (2011). Adaptation and visual coding. Journal of Vision, 11, 3–3. 10.1167/11.5.3 PMC3245980 21602298

[bibr35-03010066231100880] Webster M. A. Kaping D. Mizokami Y. Duhamel P. (2004). Adaptation to natural facial categories. Nature, 428, 557–561. 10.1038/nature02420 15058304

[bibr37-03010066231100880] Wolfe J. M. (1984). Short test flashes produce large tilt aftereffects. Vision Research, 24, 1959–1964. 6534020 10.1016/0042-6989(84)90030-0

