# Supplementary material for: A category contingent aftereffect for faces labelled with different religious affiliation is seen 7 days after adaptation
Source: Perception. 2023 May 2;52(5):297–311. doi: 10.1177/03010066231100880 (PMC10164232; doi:10.1177/03010066231100880)
Supplement: sj-docx-1-pec-10.1177_03010066231100880 - Supplemental material for A category contingent aftereffect for faces labelled with different religious affiliation is seen 7 days after adaptation [file sj-docx-1-pec-10.1177_03010066231100880.docx]

Supplemental Materials

Sample stimuli from pre-adaptation, post-adaptation (-/+ 10% distortions), and adaptation (-/+ 60% distortions) for both Christian and Muslim models.

| 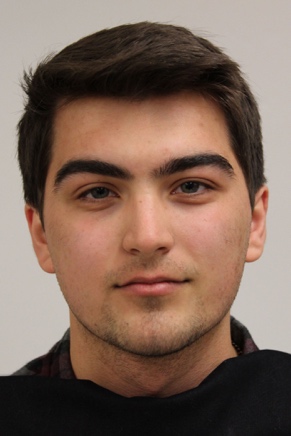  Christian, Male -10% | 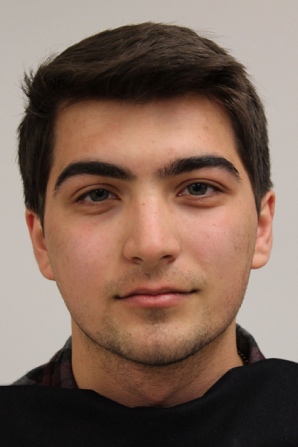  Christian, Male +10% |
| --- | --- |
| 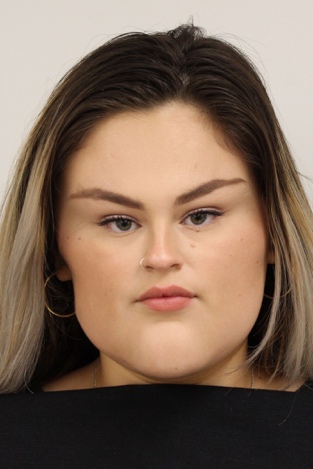  Christian, Female -60% | 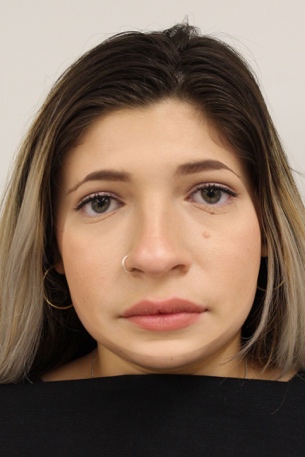  Christian, Female +60% |
| 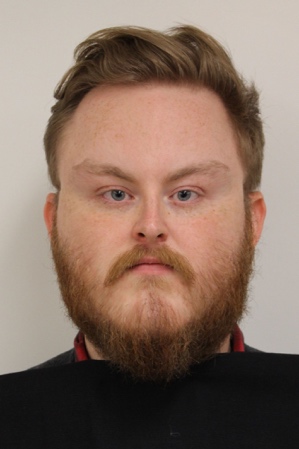  Christian, Male -60% | 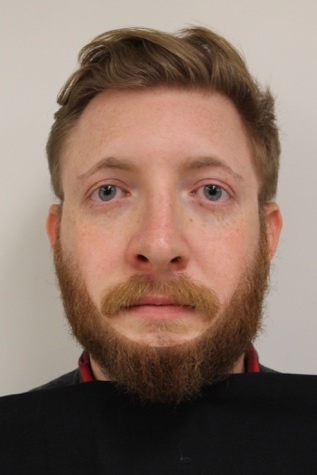  Christian, Male -+60% |
| 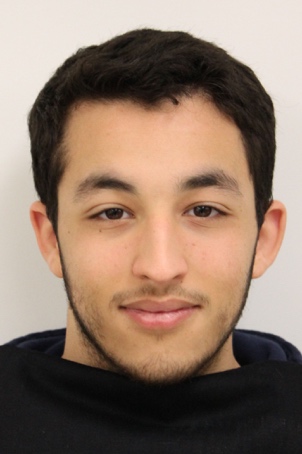  Muslim, Male -10% | 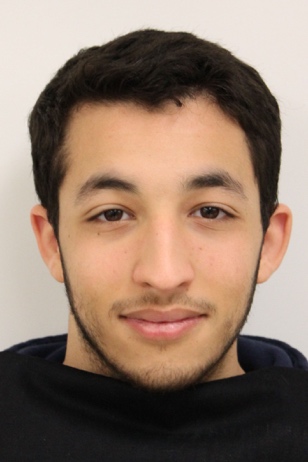  Muslim, Male +10% |
| 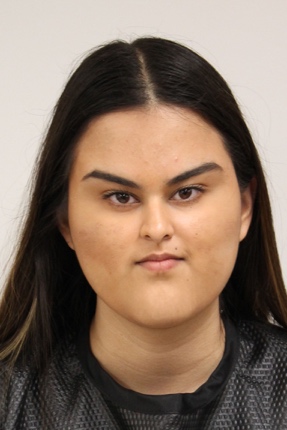  Muslim, Female -60% | 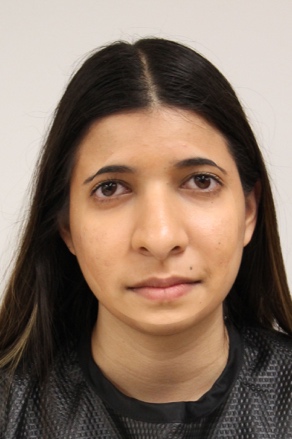  Muslim, Female +60% |
| 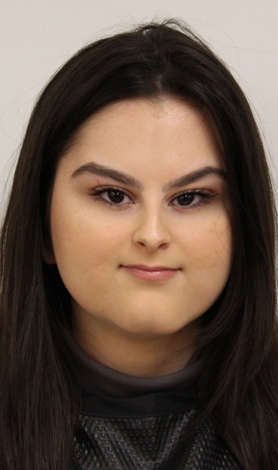  Muslim, Female -60% | 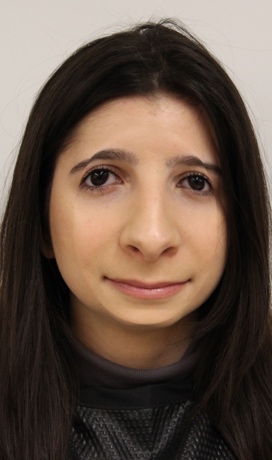  Muslim, Female +60% |
| 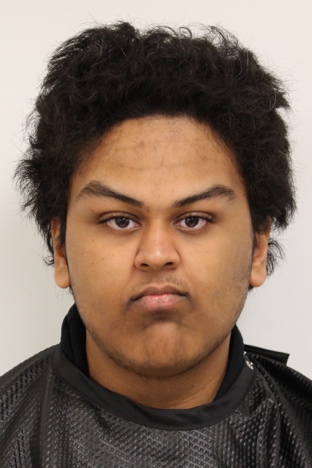  Muslim, Male -60% | 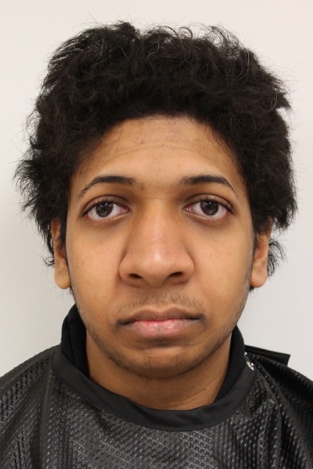  Muslim, Male -60% |
| 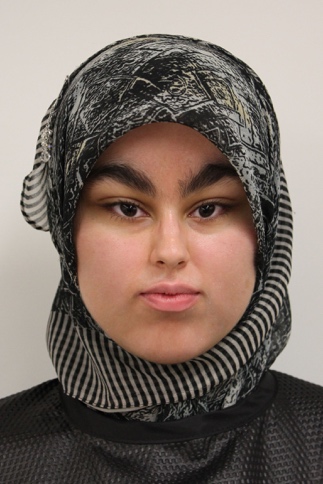  Muslim, Female -60% | 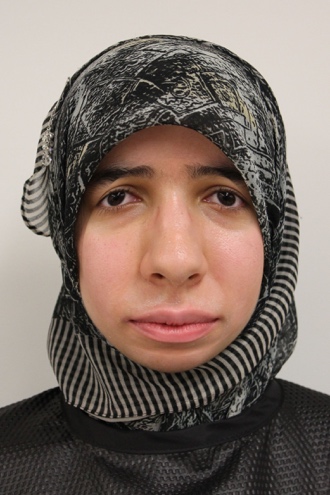  Muslim, Female +60% |
| 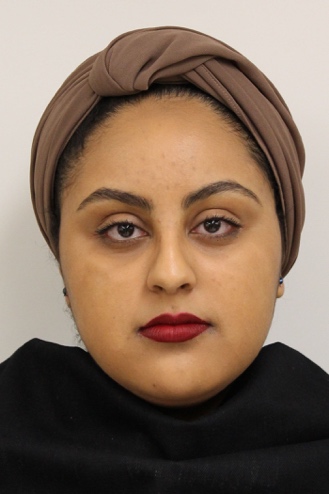  Muslim, Female -10% | 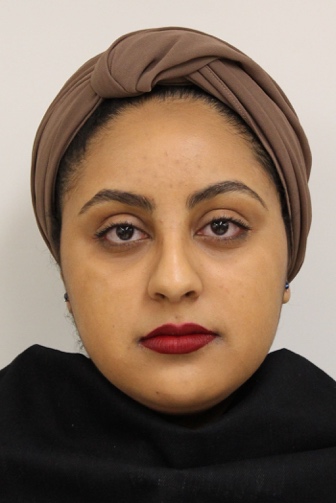  Muslim, Female -10% |
